# The Potential Role of RNA Structure in Crop Molecular Breeding

**DOI:** 10.3389/fpls.2022.868771

**Published:** 2022-05-02

**Authors:** Wenqing Sun, Ling Ding, Huakun Zhang

**Affiliations:** Key Laboratory of Molecular Epigenetics of the Ministry of Education, Northeast Normal University, Changchun, China

**Keywords:** RNA structure, translation, molecular breeding, crop improvement, riboSNitches

## Abstract

The continually growing human population creates a concomitantly increasing demand for nutritious crops with high yields. Advances in high throughput sequencing technologies have revealed the genetic architecture of major crops. This includes extensive information enabling comprehensive genetic markers for breeding selection, new gene discoveries, and novel gene regulatory strategies for crop editing. RNA structure is an important type of genetic feature, essential for post-transcriptional regulation of gene expression. Here, we summarize recent advances in genome-wide RNA structure studies in crops and review the associated RNA structure-mediated regulation of gene expression. We also discuss the functional importance of those single nucleotide variations that induce large RNA structure disparities. Lastly, we discuss the potential role of RNA structure in crop molecular breeding.

## Introduction

Crop breeding to achieve high productivity is hugely important to meet the growing demands of food sustainability and security (Hickey et al., 2019). Crop breeding strategies are based on the selection of enhanced traits among cultivars including crop yield, quality, ease of cultivation, tolerance to environmental stresses, and resistance against pathogens etc. (Breseghello and Coelho, 2013; Tian et al., 2021). Recent advances in high-throughput sequencing technologies offer a greater understanding of the genetic basis of phenotypic variations enabling precise breeding guided by the genomics (Bevan et al., 2017). Over recent decades, tremendous effort has generated vast genomics resources comprising comprehensive maps of genome variations in most major crops including rice, wheat, sorghum and tomato (Bevan et al., 2017). These genomic maps have determined millions of single-nucleotide variations (SNVs) across diverse cultivars facilitating association studies with acquired phenotypes (Huang and Han, 2014). The majority of these identified SNVs are in non-coding regions (e.g., ~97% in rice) rather than in coding regions, raising a key limitation in understanding the functions of these non-coding SNVs and thereby utilizing them to increase the genetic diversity in generating crop lines with diverse traits (Huang and Han, 2014). This poses the challenges in understanding the functions those non-coding SNVs, assisting the enhancement of genetic diversity.

Emerging evidence has shown that SNVs have the potential to alter RNA structure, offering scope for understanding the functional role of the majority of SNVs (Solem et al., 2015). Besides sequence content, RNA structure is the other key genetic property that serves an essential role in post-transcriptional regulation of processes, such as RNA degradation, translation, and splicing (Zhang and Ding, 2021). RNA structure may offer another way to interpret the results of genome-wide association studies (GWAS) (Solem et al., 2015). Recent advances in high-throughput RNA structure profiling have revealed RNA structure landscapes in crops such as rice and wheat (Deng et al., 2018; Su et al., 2018; Yang et al., 2021), not only opening novel insights into the unique RNA structural complexities in regulating gene expression in crops but also providing the potential of RNA structure-guided molecular breeding.

Here, we provide an overview of these recent discoveries of RNA structure functionalities in crops, focusing on rice and wheat. We summarize the distinctive features of crop RNA structuromes and their involvement in the post-transcriptional regulation of gene expression. We also review how crops adopted RNA structure in response to changing environmental conditions including temperature and nutrients. We emphasize the identification of SNVs in crops, known as riboSNitches, that induce large RNA structure disparities, as well as the corresponding functional impact of these SNVs. We promote discussion of how to consider adding RNA structure as a new perspective in crop breeding design.

## Unique RNA Structure Features in Crops

Recent advances in *in vivo* RNA structure profiling have generated RNA structure information over tens of thousands of genes at single nucleotide resolution (Ding et al., 2014; Rouskin et al., 2014; Spitale et al., 2015; Deng et al., 2018; Yang et al., 2021). Two types of chemical probing methods have been successfully applied in crops (Deng et al., 2018; Su et al., 2018; Yang et al., 2021). One is the dimethyl sulfate (DMS)-based method whereby single-stranded A and C are methylated by DMS (Deng et al., 2018; Su et al., 2018), whilst the other method is the SHAPE (Selective 2'-Hydroxyl Acylation analyzed by Primer Extension)-based method, whereby the single-strandedness of all four nucleotides are acetylated (Yang et al., 2021). Both *in vivo* RNA structuromes in rice and wheat captured the RNA structures for over half of their transcriptomes, providing a comprehensive view of general RNA structure features (Deng et al., 2018; Yang et al., 2021). Notably, most mRNAs *in vivo* did not fold into *in silico*-predicted structures that are thermostable RNA secondary structures, indicating *in vivo* RNA structures in crops maintained their flexibility for folding (Deng et al., 2018; Yang et al., 2021). Global structure features in rice and wheat are similar to those in *Arabidopsis* with a single-stranded region upstream of the start codon in the 5'UTR and a triplet periodic trend of RNA structure patterns across CDS regions (Ding et al., 2014; Deng et al., 2018; Yang et al., 2021).

Most major crops such as rice and wheat have high GC-content genomes and transcriptomes (Sorrells et al., 2003). Considering that RNAs with high GC content tend to fold into strong structures, one would expect that RNA structures in both rice and wheat should be very strong. Surprisingly, the high GC content in mRNAs did not lead to strong RNA structures *in vivo* (Deng et al., 2018; Yang et al., 2021). Notably, the translatome analysis in both rice and wheat revealed that mRNAs with high GC content tend to be highly translated, suggesting that RNA structures in crops might have evolved to maintain some flexibility even with high GC content to ensure active and efficient RNA biological processes such as translation (Deng et al., 2018; Yang et al., 2021).

Wheat, as a typical allopolyploid crop, contains two or more divergent genomes (subgenomes) (Yang et al., 2021). A recent study on the tetraploid durum wheat cultivar, Kronos (2*n* = 4x = 28, BBAA), found that up to 39.5% of the homoeologs between A and B subgenomes showed significantly different RNA structure features (Yang et al., 2021). Among them, 50.9% of homoeologs displayed stronger structures in the A subgenome over those in the B subgenome, while 49.1% of homoeologous pairs exhibited weaker structures in the A subgenome compared to the B subgenome (Yang et al., 2021). This asymmetry at the RNA structure level offers another layer of subgenomic diversity.

Apart from RNA secondary structure, a recent study using selective 2′-hydroxyl acylation with lithium ion-based primer extension with high-throughput sequencing (SHALiPE-seq) identified the global existence of RNA G-quadruplex structure (RG4), a specific RNA tertiary structure in rice (Yang et al., 2020). SinceRG4 plays an important role in regulating translation, it is suggested that RG4 may be one type of riboregulators across the rice transcriptome (Yang et al., 2020). Notably, rice has more RG4s with stronger folding status than *Arabidopsis* which is likely due to the high GC-content transcriptome of rice (Yang et al., 2020). Interestingly, 121 orthologous gene pairs containing RG4s that are conserved between *Arabidopsis* and rice suggest that RG4 may be strongly selected during evolution (Yang et al., 2020).

## RNA Structure-Mediated Translational Regulations of Gene Expression in Crops

With the unveiling of RNA structure landscapes in crops, new insights have been exploited in understanding the RNA structure-mediated post-transcriptional regulation of gene expression (Zhang and Ding, 2021). The translation process is one of the key post-transcriptional regulations of gene expression (Zhang and Ding, 2021). The *in vivo* RNA structure landscapes in both rice and wheat revealed that the single-stranded region upstream of the start codon in the 5′UTR was significantly associated with high translation efficiency (Deng et al., 2018; Yang et al., 2021). This single-strandedness may promote ribosome binding and/or enhance ribosome initiation. Moreover, stronger triplet periodic trends of RNA structure patterns across CDS regions were observed in those mRNAs with higher translation efficiency, indicating that the pattern might facilitate ribosome elongation (Deng et al., 2018; Yang et al., 2021). Notably, in wheat there were significant anti-correlations between average base pair probability (BPP) and translation efficiency in both 5'UTR and CDS regions, but not in 3'UTR region, suggesting that weak structures in these two genic regions enhance translation (Yang et al., 2021). This significant effect of RNA structure in regulating translation provides potential in crop editing by altering the RNA structure to obtain preferable translational levels.

**Figure 1:**
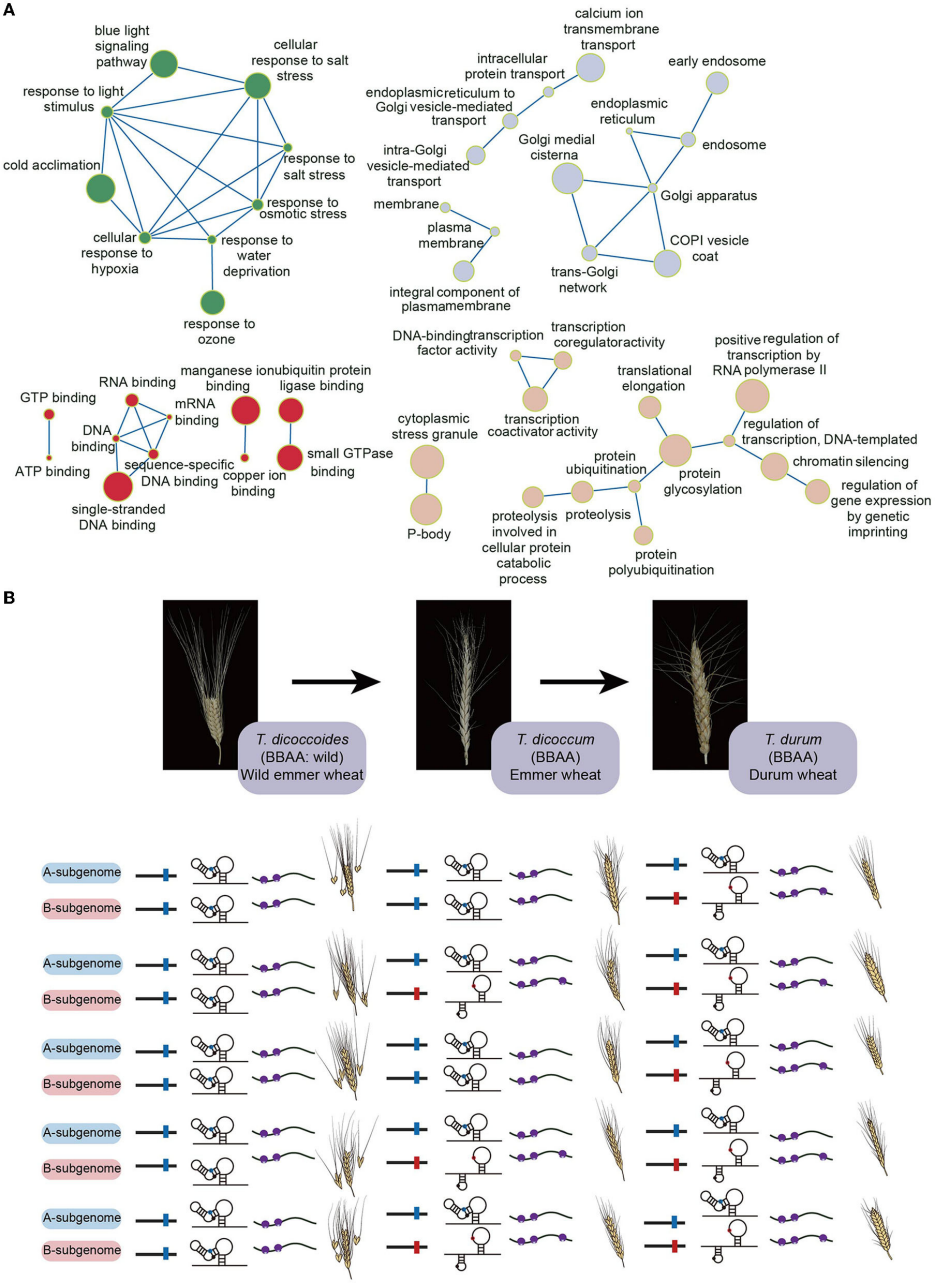
The gene functions of wheat homoeologous pairs preferring RNA structure-mediated translational subgenome asymmetry and the evolved riboSNitch during wheat domestication. **(A)** The detailed gene functions of wheat homoeologous pairs have significant correlation coefficients between subgenomic RNA structural and subgenomic translational differences (Yang et al., 2021). The green circles highlight the functions related to the responses to stimulus; the gray circles highlight the functions related to the intracellular anatomical structures; the red circles highlight the functions related to the binding functions; the rose fog circles highlight the functions related to the regulation of gene expression. The circle size represents correlation coefficients where the bigger circle size means the higher correlation coefficient. **(B)** Schematic of the differentiated riboSNitch at position 41 in the 5'UTR of the homoeologous pair *TRITD2Av1G193730* and *TRITD2Bv1g159660*. In the A subgenome homoeolog, the riboSNitch denotes the sequence of Cytosine (C41) in blue, while in the B subgenome homoeolog it denotes the sequence of Adenine (A41) in red. Small purple circles represent small ribosomal subunits, whilst big purple circles indicate large ribosomal subunits.

Recent polysome profiling in tetraploid durum wheat discovered translational subgenome asymmetry (Yang et al., 2021). Since both A and B subgenomes share a high degree of sequence similarity (~95.42%), the differences of both GC content and codon preference between A and B subgenomes are very subtle, thereby not significantly contributing to subgenomic translational difference (Yang et al., 2021). In contrast, the subgenomic RNA structure difference was significantly associated with the subgenomic translational difference (Yang et al., 2021). Notably, this relationship was much closer in those homoeologs with biological functions such as abiotic stress response, biotic stress response, metal ion response, phytohormone signaling, transcriptional and translational regulations (Figure 1A) (Yang et al., 2021). Therefore, compared to GC content and codon preference, RNA structure may play a more prevalent role in regulating translational subgenome asymmetry (Yang et al., 2021).

Apart from transcriptome-wide studies, an antisense-mediated translational regulation has been discovered in rice. The antisense RNA (*cis-NATpho1.2*) interacts with its sense RNA, the phosphate transporter *PHOSPHATE1.2* (*PHO1.2*) at a high GC region across the *PHO1.2* start codon (Reis et al., 2021). This sense-antisense inter-molecular interaction rearranged the local structure, allowing the access of 60S to the translation initiation site for the formation of 80S, thereby enhancing translation (Reis et al., 2021). Interestingly, the expression of *cis-NATpho1.2* was increased under phosphate starvation conditions (Jabnoune et al., 2013). The translation of *PHO1.2* was correspondingly enhanced in facilitating phosphate transport from roots to shoots (Jabnoune et al., 2013). This antisense-mediated translational regulation via RNA structure rearrangement was proposed to be an RNA structure nutrient switch in crop response to different levels of phosphate in the soil (Reis et al., 2021).

## RNA Structure-Mediated Degradation in Crops

The folding status of RNA is crucial in determining its thermodynamic properties, thereby its stability (Bevilacqua et al., 2016). Temperature is reported as one of the key factors affecting RNA structure (Bevilacqua et al., 2016). A recent study of *in vivo* RNA structure in rice using the DMS-based method found that RNA structure is globally unfolded in response to heat stress at 42°C and where the 3′UTR region showed the greatest response (Su et al., 2018). This change of RNA structure at 42°C was significantly associated with the decrease of RNA abundance, indicating the promotion of RNA degradation (Su et al., 2018). Through sequence content investigation, those transcripts with both 5'UTR and 3'UTR enriched with AU content tended to reduce their RNA abundance more in response to heat stress (Su et al., 2018). This is likely due to the weaker AU base-pairing being easier to disrupt under high temperature vs. GC. Surprisingly, although there were significant changes of RNA structure in 5'UTR and across the start codon, the translation efficiency did not alter correspondingly (Su et al., 2018), and this is very different from the RNA structure in response to temperature in prokaryotes (Krajewski and Narberhaus, 2014). Alternatively, this may be due to using a short period of heat treatment. The global RNA structure alteration in response to heat stress in rice provides evidence of RNA structure-mediated gene regulation in response to environmental stress. With global warming seriously threatening global food security (Lesk et al., 2016), this RNA structure-mediated regulation of gene expression in response to heat stress could open new perspectives for crop improvement.

## SNVs Are Capable of Changing RNA Structure-Mediated Regulations of Gene Expression in Crops

RNA folding status largely relies on sequence content (Mathews et al., 2010). Therefore, a single nucleotide variant (SNV) is capable of altering RNA structure (Solem et al., 2015). Those SNVs resulting in RNA structural rearrangements are termed riboSNitches (Solem et al., 2015). Emerging evidence of individual riboSNitches alongside the discovery of riboSNitches in human transcriptomes suggested the importance of riboSNitches for interpreting GWAS results for human genetics disease (Wan et al., 2014; Solem et al., 2015; Corley et al., 2017). Recent studies in crops also raise the potential in utilizing riboSNitches for molecular breeding.

One of the studies in rice found that a 1,236 nt long non-coding RNA, referred to as long-day-specific male-fertility–associated RNA (LDMAR) regulated photoperiod-sensitive male sterility (Ding et al., 2012). A spontaneous mutation causing an SNV (G–C) between the wild-type and mutant disrupted the base pairs in the stem, resulting in an internal bulge based on *in silico* RNA structure prediction (Ding et al., 2012). This local RNA structure on the LDMAR led to the reduction of its transcriptional level, subsequently causing photoperiod-sensitive male sterility (Ding et al., 2012). It was suggested that this structure change might affect the function of LDMAR indirectly via DNA methylation (Ding et al., 2012). Although the detailed mechanism of this SNV-induced RNA structure change remains elusive, requiring *in vivo* RNA structure analysis, the impact of RNA structure on rice photoperiod regulation is of great significance, opening prospects for developing male-sterile germplasm for hybrid crop breeding.

Four genes (*VRN1*-*VRN3* and *VRN-D4*) were previously identified for regulating vernalization in wheat (Kippes et al., 2015). Inside the *VRN1* first intron, there were binding sites for the RNA-binding protein *GLYCINE-RICH RNA-BINDING PROTEIN 2* (*TaGRP2*) (Xiao et al., 2014), homologous to the *Arabidopsis GLYCINE-RICH RNA-BINDING PROTEIN 7* (*GRP7*) that is a single-stranded RNA-binding protein involved in the regulation of flowering (Streitner et al., 2008). Under normal conditions, TaGRP2 binds to *VRN1* pre-mRNA and inhibits *VRN1* expression (Kippes et al., 2015). In contrast, three adjacent SNVs inside the *VRN-D4* first intron enlarged the end loop thereby disrupting TaGRP2 binding, based on *in silico* RNA structure prediction (Kippes et al., 2015). Interestingly, *VRN-D4* was found in most accessions of the ancient subspecies *Triticum aestivum* ssp. *sphaerococcum* from South Asia, indicating *VRN-D4* may contribute to local adaptation (Kippes et al., 2015). Further *in vivo* RNA structure probing could provide more detailed mechanistic insights on the RNA structure-mediated regulation of the interaction between RNA and RNA binding proteins, that may be applied in modulating vernalization and developing wheat varieties better adapted to local habitats.

In addition to these individual examples of the importance of SNV-induced RNA structure alteration, a recent genome-wide study on the *in vivo* RNA structure landscape in the tetraploid durum wheat cultivar, Kronos, identified 3,564 SNVs which induced large structure disparities, known as riboSNitches between the A and B subgenomes (Yang et al., 2021). Among these riboSNitches, there were more transition (i.e., similar shape base interchanges) riboSNitches than transversion (i.e., dissimilar shape base interchanges) riboSNitches (Yang et al., 2021). These riboSNitches tend to be conserved across tetraploid *T. turgidum* spp. *durum*, suggesting their importance on RNA structures and corresponding functions.

Durum wheat (*T. turgidum* ssp. *durum*) is a major cereal grain used for pasta and couscous production, evolved from domesticated emmer wheat (*T. turgidum* ssp. *Dicoccum*). Domesticated emmer wheat was derived from wild emmer wheat (*T. turgidum* ssp. *Dicoccoides*) in the Fertile Crescent about 10,000 years ago (Maccaferri et al., 2019). During domestication across these three sub-species, riboSNitches located in both 5'UTR and 3'UTR were under stronger selection pressure compared to non-riboSNitches (Yang et al., 2021). One differentiated riboSNitch at position 41 in the 5'UTR of homoeologous pair *TRITD2Av1G193730* and *TRITD2Bv1g159660* (annotated as ribosomal protein L11 family proteins) was used to demonstrate its effect on molecular functions (Yang et al., 2021). In the A subgenome homoeolog, the riboSNitch denotes the sequence of Cytosine (C41), while in the B subgenome homoeolog it denotes the sequence of Adenine (A41) (Yang et al., 2021). This riboSNitch remains the same across all durum wheat accessions, with C41 in the A subgenome and A41 in the B subgenome (Yang et al., 2021). In contrast, the nucleotide frequency of the B subgenome A41 nucleotide in all the domesticated emmer wheat accessions is 72% whilst 95% of wild emmer wheat accessions harbor C41 (Yang et al., 2021). This C41/A41 riboSNitch resulted in significant RNA structure differences between A and B subgenome homoeologs (Yang et al., 2021). The C41 formed into a C-G base pair with G22 in the A subgenome homoeolog while the A41 is single-stranded in the B subgenome homoeolog (Yang et al., 2021). Notably, this riboSNitch caused the translation efficiency of the A subgenome to be significantly lower than that of the B subgenome, suggesting that this riboSNitch was selected during domestication to diversify the translation efficiency between subgenomes (Yang et al., 2021). Notably, the annotated gene function of this homoeologous pair is related to the translation. Thus, this riboSNitch might evolve to regulate the subgenomic function in translation. Based on this example, it is likely that the utilization of riboSNitches during evolution is parallel to or earlier than amino acid evolution, supporting the RNA world theory (Hirao and Ellington, 1995; Robertson and Joyce, 2012). Therefore, the scope for editing RNA structure divergence between subgenomes offers potential in accelerating domestication (Figure 1B).

## Future Perspectives

**Figure 2:**
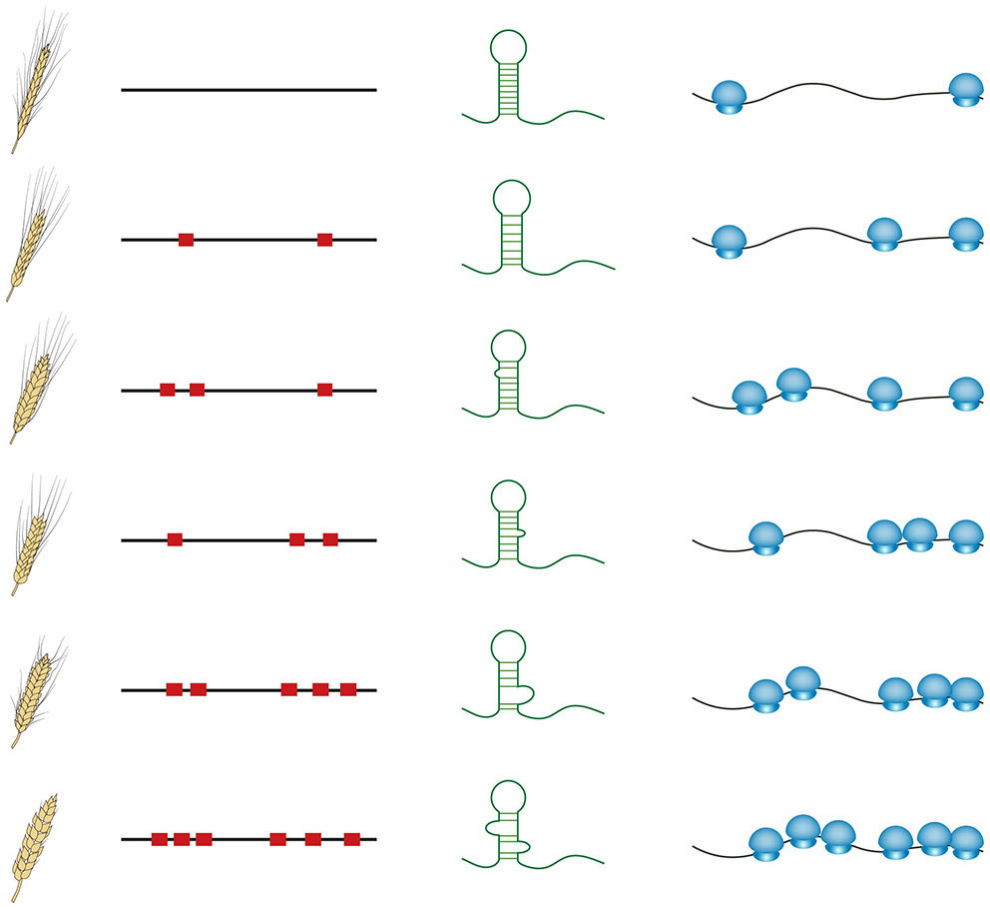
Schematic of the diversity among phenotypes, genome sequences, RNA structures, and translational levels across natural varieties. Red squares represent the sequence variations. Small blue circles represent small ribosomal subunits, whilst big blue circles indicate large ribosomal subunits.

These recent studies provide evidence that RNA structure could serve as an important genetic property to guide crop breeding. In contrast to other cis-regulatory motifs such as transcription factor binding sites, influencing transcriptional levels, RNA structure features mostly affect post-transcriptional regulation of gene expression, directly impacting protein synthesis (Gebauer and Hentze, 2004; Zhang and Ding, 2021). The sequence diversity in numerous natural varieties is likely to cause RNA structural diversity, subsequently causing different translational levels (schematic in Figure 2). Comprehensive *in vivo* RNA structure profiling and translatome analysis across these natural varieties should be able to explain a large proportion of genomic sequencing diversity, particularly those in non-coding regions. Furthermore, genome association studies could be conducted among the phenotypes, the genomes, RNA structure features, and translational levels to elucidate the molecular mechanism of RNA structure-mediated translation underlying associations between phenotypes and genotypes (Figure 2). Clear mechanistic understanding will enable a more accurate selection of breeding lines to achieve preferable translational levels and thereby appropriate protein levels. With the application of clustered regularly interspaced short palindromic repeats (CRISPR) genome editing technologies in crops (Zhang et al., 2018), precise editing specifically targeting those functional RNA structure regions will be able to directly generate the ideal RNA structure in transgenic crops with preferable protein levels.

It was proposed that the subgenome asymmetry in wheat was evolved in control of both vital genes and genes involved in environmental adaptations (Feldman et al., 2012). Interestingly, the biological functions of mRNAs that preferred the RNA structure-mediated translational subgenome asymmetry include abiotic stress response, biotic stress response, metal ion response and phytohormone signaling, and transcriptional and translational regulations (Figure 1A). Therefore, maximizing the translational subgenomic asymmetry is possible by increasing the RNA structure difference between subgenomes for those vital and environmental responsive genes, offering a new wheat editing and breeding strategy.

The discovery of riboSNitches selected during wheat domestication also offers scope for innovative crop breeding. In the riboSNitch example illustrated above (Figure 1B), the sequences of the homoeologous pair in the wild emmers are the same between A and B subgenomes that lead to the same subgenomic RNA structures, thereby the same subgenomic translational efficiencies (Figure 1B). In contrast, the sequence divergences between A and B subgenomes in the domesticated cultivars of durum wheat populations cause differentiated RNA structures, and subsequent different translational efficiencies (Figure 1B). This riboSNitch could serve as a molecular marker during the selection of breeding lines. Additionally, if this subgenomic divergence was selected during domestication, nucleotide-editing CRISPR technology could precisely modify the RNA structures to obtain greater divergences between A and B subgenomes, leading to a greater differentiation of translational subgenome expression asymmetry, that may offer a strategy for accelerating the domestication process.

Adaptation to changing environmental conditions such as abiotic stress and biotic stress is essential to crop growth. Most environmental factors such as temperature, heavy metals, nutrients, salt, and light affect RNA folding (Chung et al., 2020; Zhang and Ding, 2021; Zhu et al., 2021). Thus, further work in understanding RNA structure alterations in plant response to varying environmental conditions is of great importance in developing crop lines with high environmental fitness to achieve sustainable crop growth and food production. Moreover, building on the diversities of genomes and phenotypes, future genome association studies could focus on exploiting RNA structure diversity in response to changing environmental conditions. The outcomes could offer guidance for breeding crop lines that demonstrate robust environmental tolerances.

## Author Contributions

WS, LD, and HZ wrote the manuscript together. All authors contributed to the article and approved the submitted version.

## Funding

HZ was supported by the National Natural Science Foundation of China (32170229) and the National Key Research and Development Program of China (2021YFF1000900).

## Conflict of Interest

The authors declare that the research was conducted in the absence of any commercial or financial relationships that could be construed as a potential conflict of interest.

## Publisher's Note

All claims expressed in this article are solely those of the authors and do not necessarily represent those of their affiliated organizations, or those of the publisher, the editors and the reviewers. Any product that may be evaluated in this article, or claim that may be made by its manufacturer, is not guaranteed or endorsed by the publisher.
